# Microfluidic manufacturing of different niosomes nanoparticles for curcumin encapsulation: Physical characteristics, encapsulation efficacy, and drug release

**DOI:** 10.3762/bjnano.10.177

**Published:** 2019-09-05

**Authors:** Mohammad A Obeid, Ibrahim Khadra, Abdullah Albaloushi, Margaret Mullin, Hanin Alyamani, Valerie A Ferro

**Affiliations:** 1 Department of pharmaceutical sciences, Faculty of Pharmacy, Yarmouk University, Irbid, Jordan; 2 Strathclyde Institute of Pharmacy and Biomedical Sciences, University of Strathclyde, 161 Cathedral Street, G4 0RE Glasgow, United Kingdom; 3 Oman College of Health Sciences, School of Pharmacy, Muscat, Oman; 4 Institute of Infection Immunity and Inflammation, College of MVLS, University of Glasgow, Glasgow, United Kingdom

**Keywords:** curcumin, drug delivery, microfluidic mixing, niosomes nanoparticle

## Abstract

Curcumin, a natural chemical compound found in *Curcuma longa* that has been used in antitumor and anti-inflammation applications, exhibits very limited water solubility and rapid in vivo degradation, which limits its clinical application. To overcome these limitations, niosome nanoparticles were prepared by microfluidic mixing for curcumin encapsulation. Niosome nanoparticles are lipid-based, and composed of non-ionic surfactants with cholesterol orientated into a membrane bilayer structure. Two different non-ionic surfactants were used and the mixing parameters were varied to optimize the characteristics of the prepared niosomes. The prepared niosomes had an average particle size of 70–230 nm depending on the type of non-ionic surfactant used and the mixing parameter. Moreover, all prepared niosomes were monodisperse with an average polydispersity index ranging from 0.07 to 0.3. All prepared niosomes were spherical as demonstrated by transmission electron microscopy. Curcumin was encapsulated with a maximum encapsulation efficiency of around 60% using Tween 85 as the non-ionic surfactant. Niosomes prepared by microfluidic mixing provided a controlled release of curcumin, as indicated by the release profile of curcumin, improving its therapeutic capability. These results demonstrate that niosomes prepared by microfluidic mixing to encapsulate curcumin are a promising delivery system to reach target cells.

## Introduction

Curcumin is a natural product that is derived from the rhizome of the medicinal plant *Curcuma longa Linn* [1]. It has different therapeutic applications such as the use against inflammation and respiratory distress [2]. Moreover, in several studies, curcumin has been proven to have chemopreventive and chemotherapeutic effects against several types of cancer such as prostate and cervical cancers [3]. However, the therapeutic application of curcumin is limited by its high hydrophobicity with poor water solubility, photosensitivity, chemical instability, and rapid metabolism rate [4–5]. Therefore, as a result, systemic bioavailability is much reduced [6]. The use of nanoparticles as drug delivery systems is currently a corner stone in the field of drug delivery in order to improve the pharmacokinetics and pharmacodynamics of many drugs that have limitations in bioavailability [7]. Therefore, to improve the curcumin characteristics, nanoparticles have been proposed as carriers for curcumin to enhance its distribution and permeability [4]. Different types of nanoparticles have been investigated for curcumin delivery in order to prolong the plasma circulation time and enhance the localization of the drug in the target tissues while reducing the unwanted side effects [8–9]. Liposomes, solid lipid nanoparticles, dendrimers, micelles, polymeric nanoparticles, gold nanoparticles, and carbon nanotubes are among the most common types of nanoparticle delivery systems [10].

These efforts have been reported in several studies. For example, Guo et al. were able to efficiently encapsulate curcumin into polymeric nanoparticles prepared using a fabricated microchannel. The prepared polymeric nanoparticles had an average particle size of 167 nm with a curcumin loading capacities of 15% [11]. Using niosome nanoparticles composed of different non-ionic surfactants prepared by the solvent evaporation method, Xu et al. were able to achieve around 92% loading efficiency of curcumin and measured enhanced cytotoxic activity against ovarian cancer cells compared with freely dispersed curcumin [9]. Microfluidic mixing is a recently developed method for the preparation of niosomes that allows for the control of particle size and polydispersity, without the need for a size-reduction step after the particles preparation [12]. Niosomes can be prepared within the required characteristics in a single step, which can be later used for large industrial scale preparations [13].

In the present work, niosomes for curcumin encapsulation were prepared by microfluidic mixing. Microfluidic mixing is a fast and reliable method for the preparation of niosomes, which allows for the preparation of small and monodisperse particles within seconds. Different formulations encapsulating curcumin were prepared by microfluidic mixing by varying the surfactants and mixing parameters. Previously, our lab successfully developed empty niosomes through microfluidic mixing using different types of surfactants such as Tween 85 or Span 85 at different ratios. In this work, these surfactants have been used to examine the efficiency of the prepared niosomes in curcumin encapsulation. The physicochemical characteristics were assessed and the ability of the niosomes to encapsulate and then release the loaded curcumin was evaluated.

## Experimental

### Materials

Sorbitan monooleate (Span 80, SP80), polyoxyethylenesorbitan trioleate (Tween 85, T85), cholesterol (Chol), curcumin, ethanol, methanol, and cellulose membrane with molecular weight cut-off = 14000 were purchased from Sigma-Aldrich (UK).

### Preparation of SP80 and T85 niosomes by microfluidic mixing

Niosomes composed of SP80 or T85 as a surfactant with Chol were prepared using microfluidic mixing on a NanoAssemblrTM (Benchtop, Precision NanoSystems Inc., Vancouver, Canada) as described previously [12]. The mixing process takes place in a microfluidic cartridge with staggered herringbone structures, which has two inlets, one for the organic phase and the other for the aqueous phase. The organic phase was prepared by dissolving the lipid components (Sp80 or T85 with Chol at a 50:50 molar ratio) with or without curcumin in ethanol while the aqueous phase was deionised water. The mixing process carried out at 50 °C using a heating block. Both phases were injected into the microchannel using disposable syringes through syringe pumps. Niosomes were prepared at 1:1 and 3:1 flow rate ratios (FRR) between the aqueous and the lipid phases and all formulations were prepared at a total flow rate of 4 mL/min. The initial curcumin concentrations were 410 µg/mL and 210 µg/mL for formulations prepared at 1:1 and 3:1 FRR, respectively.

### Removal of the non-encapsulated drug

Non-encapsulated curcumin was removed by dialysis against ten times the volume of deionised water under continuous stirring at room temperature. At different time points, 1 mL was taken from the dialysis media and the amount of curcumin was measured using UV absorption spectroscopy at 421 nm using a HELIOS ALPHA ThermoSpectronic spectrophotometer (Thermo Fisher Scientific, UK). The curcumin concentration was determined using a calibration curve of the pure drug in methanol. After removal of each sample, 1 mL of deionised water was added to the dialysis media to maintain sink conditions. The dialysis was carried out until a constant curcumin concentration was detected in the dialysis media.

### Physicochemical characterisation of niosomes

#### Particle size analysis

The average particle size (*Z*_Average_) and the PDI of the niosomes with and without the curcumin was measured by dynamic light scattering (DLS) using a Zetasizer Nano-ZS (Malvern Instruments Ltd., UK). All the samples were diluted at 1/20 using deionised water and the measurements in triplicate were taken at 25 °C.

#### Niosome morphology

The morphological examination of the prepared niosomes was determined using transmission electron microscope (TEM). Briefly, carbon-coated copper grids (400 mesh, agar scientific) were glow discharged in air for 30 seconds. Sample solution (3 µL) was drop-cast on the grids and were then negatively stained using uranyl acetate. Each sample was allowed to dry afterwards in a dust-free environment prior to TEM imaging. The dried samples were then imaged using a JEOL JEM-1200EX TEM (JEOL, Tokyo, Japan) operating at an accelerating voltage of 80 kV.

#### Determination of curcumin encapsulation efficiency

Following removal of the non-encapsulated curcumin by dialysis, 100 µL of each niosome formulation (removed from the dialysis tubing) was lysed with methanol in order to release the encapsulated curcumin, which was then quantified at UV absorbance at 421 nm. The encapsulation efficiency (EE) of curcumin was determined according to the following equation:


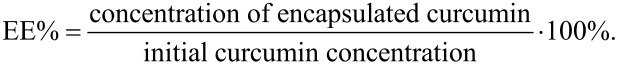


The experiments were performed in triplicate and the mean ± SD is reported.

#### In vitro release profile of curcumin

After removal of the non-encapsulated curcumin, 3 mL of each formulation loaded with curcumin were placed in separate dialysis tubes and dialysed against ten times the volume of deionised water, at 37 °C under continuous stirring. Samples were taken every day for a total of 21 days. At each time point, 1 mL from the dialysis media from each niosomes formulation was taken and replaced with fresh 1 mL deionised water preheated at 37 °C. At each time point, the absorbance was measured at 421 nm and the concentration of the released curcumin was determined against a calibration curve. The experiments were performed in triplicate and the mean ± SD is reported.

### Statistical analysis

Statistical significance was assessed by one-way analysis of variance (ANOVA) and Tukey multiple comparison test and *t*-test was performed for paired comparisons using Minitab^®^ software, State College, PE. Differences were considered statistically significant for *p* < 0.05.

## Results and Discussion

### Preparation of niosomes using microfluidic mixing

Two types of surfactant (SP80 and T85) with chol were used to prepare niosomes. The aim was to see the effect of changing the surfactant type on the encapsulation of curcumin. Each formulation was prepared at two different FRR between the aqueous and the lipid phases during the microfluidic mixing. Table 1 shows the particle sizes calculated using DLS for the niosome formulations. At 1:1 FRR, changing the surfactant type from SP80 to T85 resulted in a significant (*p* < 0.05) increase in the average particle size and distribution for both empty and loaded particles. However, this was not the case at 3:1 FRR where the particle sizes and distributions were the same for both surfactant types. As can be seen from Table 1, the change in the FRR from 1:1 to 3:1 for the same noisome formulation resulted in a significant (*p* < 0.05) decrease in particle size and distribution. For example, the size of the empty SP80 niosomes decreased significantly (*p* < 0.05) from ca. 142 to ca. 70 nm by increasing the FRR from 1:1 to 3:1. These results confirm our previous studies describing niosome preparation by microfluidic mixing the same types of surfactants [12,14].

**Table 1:** Size and distribution for niosomes prepared by microfluidic mixing with two types of non-ionic surfactants at two different FRR. Results represent the mean ± SD of triplicate readings.

Sample	Size of empty particles (nm)	PDI of empty particles	Size of loaded particles (nm)	PDI of loaded particles

SP80 1:1	142.30 ± 1.05	0.14 ± 0.05	144.50 ± 1.84	0.17 ± 0.05
SP80 3:1	70.51 ± 0.43	0.11 ± 0.04	70.26 ± 0.20	0.09 ± 0.02
T85 1:1	228.33 ± 17.56	0.33 ± 0.04	231.75 ± 22.70	0.34 ± 0.03
T85 3:1	71.31 ± 0.70	0.07 ± 0.01	75.41 ± 1.03	0.09 ± 0.02

### Niosome morphology

**Figure 1:**
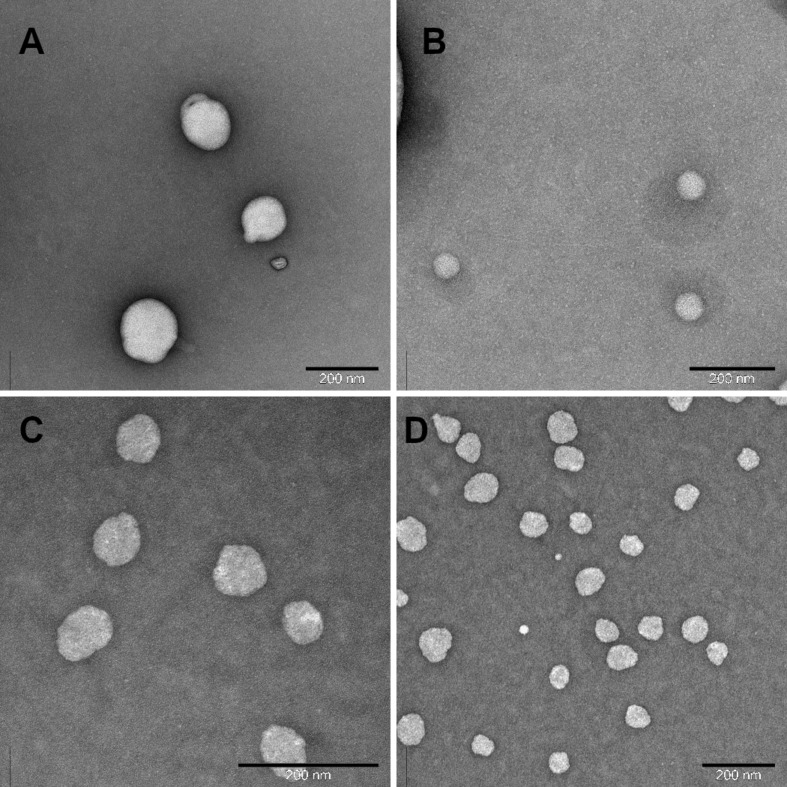
TEM images of (A) SP80 niosomes prepared at 1:1 FRR, (B) SP80 niosomes prepared at 3:1 FRR, (C) T85 niosomes prepared at 1:1 FRR, (D) T85 niosomes prepared at 3:1 FRR.

TEM was used to examine the morphological characteristics of the niosomes and the results are illustrated in Figure 1. The results indicated that the prepared niosomes were almost spherical with diameters matching the results obtained from DLS. Moreover, the TEM images clearly confirm the effect of changing the FRR on the particle sizes, where smaller particles were obtained at a ratio of 3:1 compared to a ratio of 1:1 for both formulations. These results confirm previously reported results about niosomes prepared by microfluidic mixing [15].

### Encapsulation of curcumin

**Figure 2:**
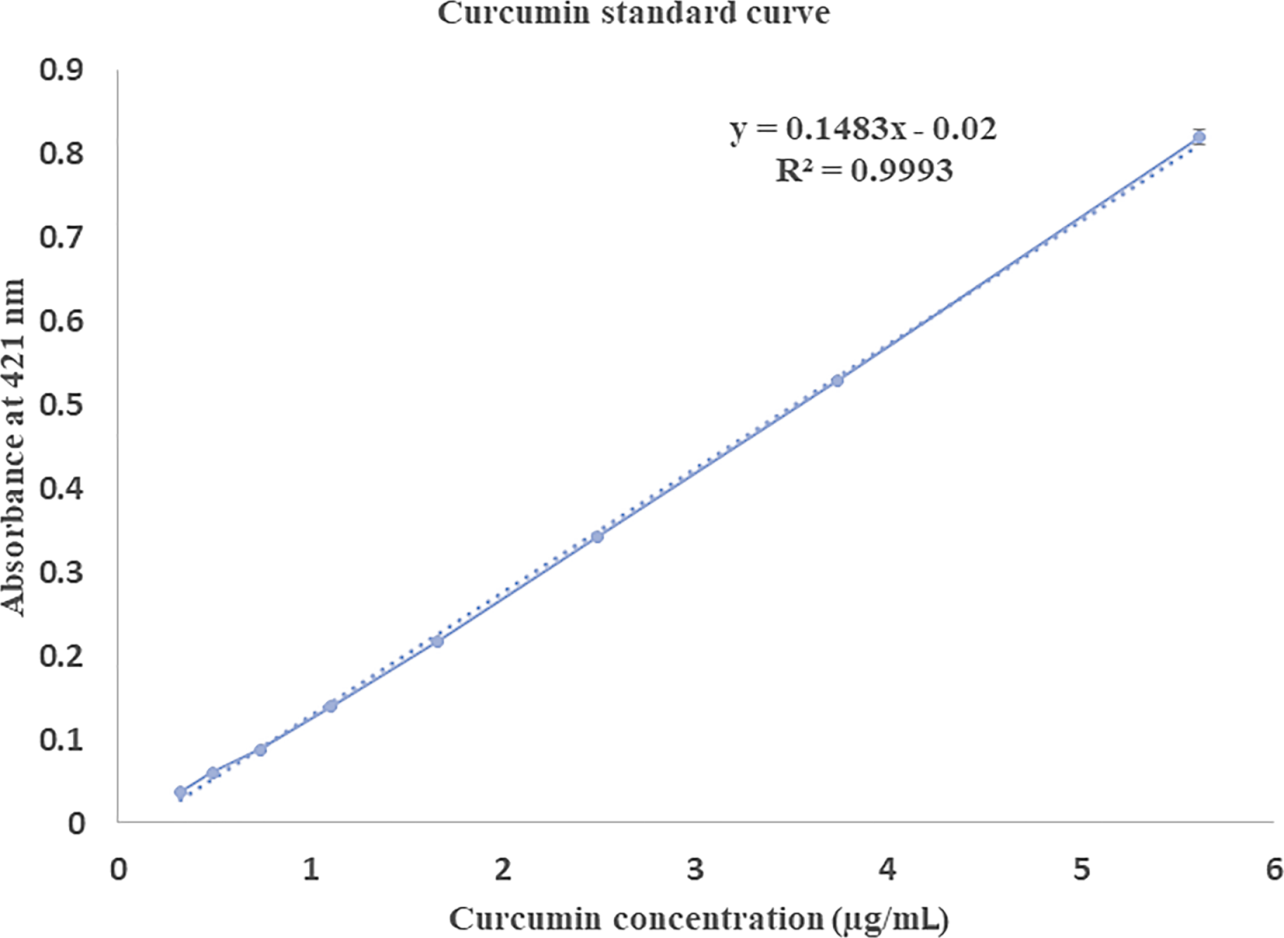
Curcumin standard curve measured by UV spectroscopy at 421 nm.

The potential of a nanoparticle delivery system can be predicted based on its encapsulation efficiency (EE) values. The EE data for the two niosome preparations from two different FRR was determined using a curcumin standard curve (Figure 2) as shown in Table 2. The use of T85 resulted in a significantly (*p* < 0.05) higher encapsulation of curcumin compared to niosomes prepared using SP80 at the same FRR. Here at the same FRR, the only factor that was changed is the type of the non-ionic surfactant and this had a significant impact on the final curcumin encapsulation. For example, niosomes prepared with SP80 at 3:1 FRR resulted in a curcumin EE of ca. 11%, while niosomes prepared using T85 at 3:1 FRR resulted in an EE of ca. 60%. The increase in FRR from 1:1 to 3:1 resulted in a significant (*p* < 0.05) increase in the EE values for both formulations.

**Table 2:** Encapsulation efficiency (%EE) for curcumin using niosomes prepared with two different non-ionic surfactants at two different FRR. Results represent the mean ± SD of triplicate readings.

Sample	Curcumin %EE

SP80 1:1	1.21 ± 0.03
SP80 3:1	10.59 ± 0.05
T85 1:1	9.57 ± 0.02
T85 3:1	59.45 ± 0.20

Niosomes exhibit a lipid bilayer structure encapsulating an aqueous compartment. Hydrophilic molecules are encapsulated in the aqueous compartment, while hydrophobic molecules are embedded in the membrane [16]. The EE of hydrophobic molecules is majorly affected by the lamellarity of the niosome membranes. Because the niosomes discussed here are unilamellar, the main factor that seems to affect the EE of the niosomes is the FRR and, consequently, the particle size, because higher FRR values resulted in a smaller particles and higher EE. This can be explained by the difference in the final chol concentrations when changing the FRR from 1:1 to 3:1. The increase in the FRR results in a lower concentration of chol and the non-ionic surfactant in the final niosome formulations. The presence of chol is a key factor that controls membrane rigidity. Higher chol concentrations results in a less permeable and more rigid bilayer membrane. Moreover, the encapsulation of hydrophobic molecules is highly dependent on the bilayer membrane fluidity where higher encapsulation values can be achieved with less rigid membranes [17]. In addition, higher chol concentrations might compete with the hydrophobic molecules and prevent its encapsulation during the self-assemble of the lipid components into the bilayer structure [18]. Based on that, the increase in the FRR from 1:1 to 3:1 will result in a lower chol concentration in the final preparation, which means less rigid membranes and higher encapsulation values for hydrophobic curcumin. This explains the observed EE results.

Gupta et al. prepared niosomes composed of Span 60 and chol at a 70:30 molar ratio using the reverse evaporation method for nanoparticle preparation and achieved around 68% curcumin EE [19], which is comparable with our niosomes preparation using T85 at 3:1 FRR. Similarly, Manca et al., achieved curcumin EE of about 66% using liposomes containing the polyanion sodium hyaluronate [8]. Ozeki et al., prepared a curcumin-loaded PEGylated PLGA through microfluidic mixing and achieved an EE of around 50% [20]. Here, high EE of around 60% was achieved using niosomes nanoparticles composed of T85 as a non-ionic surfactant.

The encapsulation efficiency of the niosomes nanoparticles depends on several factors related to the characteristics of the non-ionic surfactant and the molar ratio between surfactant and cholesterol [21]. It has been reported that the size of the hydrophilic head group, the chain length of the non-ionic surfactant, the hydrophilic–lipophilic balance (HLB), and the phase transition temperature (*T*_c_) of the surfactant in the niosomes formulation would significantly affect the encapsulation efficiency for different drugs [22]. Here, two different types of non-ionic surfactants were used in the preparation of niosomes for curcumin encapsulation and since these two surfactants have different characteristics, this would explain the differences in the EE of curcumin between the prepared formulations.

### In vitro release profile of curcumin

**Figure 3:**
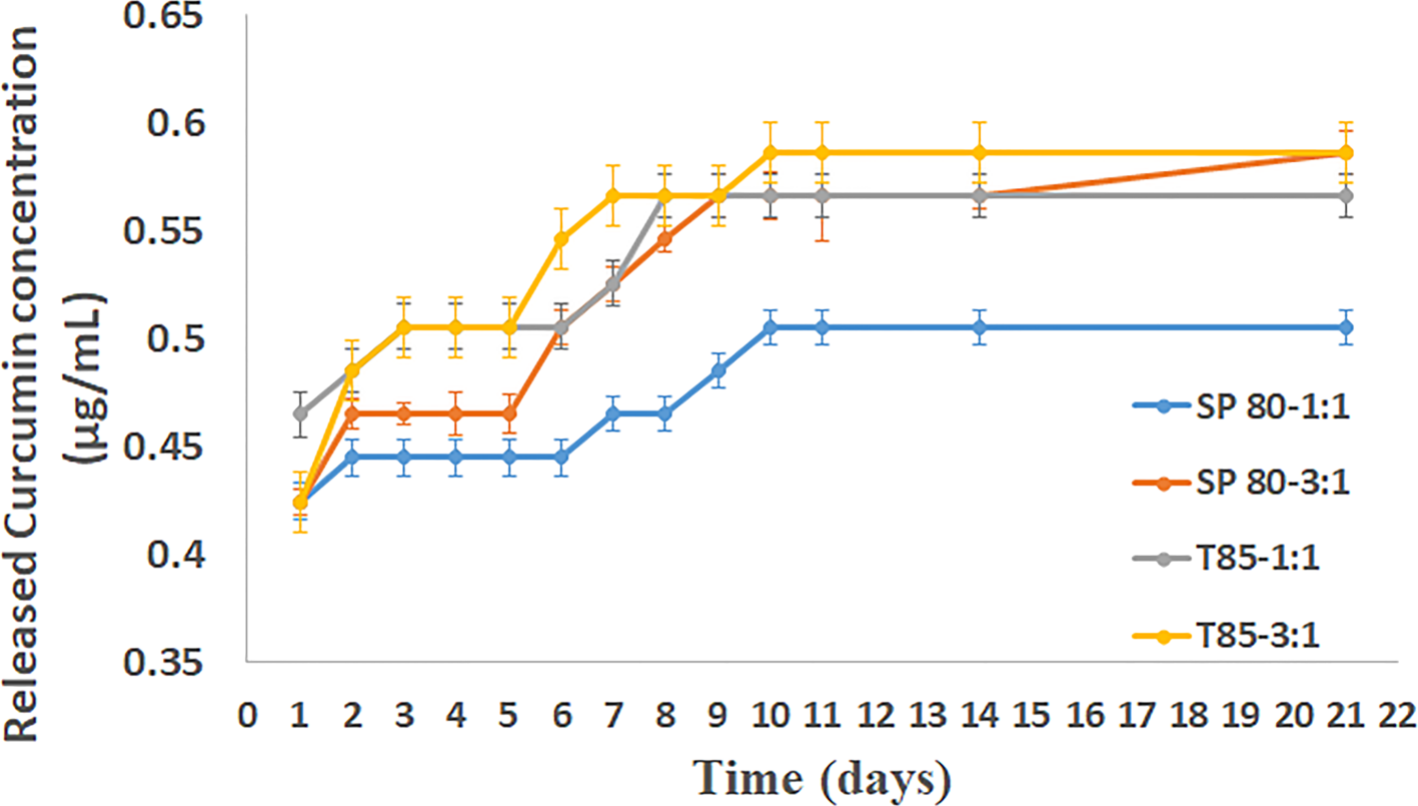
Curcumin release profile from the different niosome preparations when stored at 37 °C.

The in vitro release of curcumin from the niosomes is shown in Figure 3 and exhibited a biphasic pattern where an initial burst occurred followed by constant release and then another release pattern began followed by constant release for all the niosome preparations. The burst release rate reached a maximum within two days of storage and then the curcumin release concentration was constant until day 5 where an increase then followed.

These results suggest that the niosomes provide excellent release of curcumin as indicated by Figure 3. The release profile from all the formulations was almost identical despite having different EE. For example, the release profile of the T85 niosomes prepared at 3:1 FRR, which had the highest EE of around 60%, was the same as that of the other niosome formulations. All the prepared niosomes in this study had the same percentage of chol for membrane stability purposes [18], which is probably a contributing factor to the release profile being the same.

## Conclusion

The preparation and characterization of niosomes nanoparticles prepared by microfluidic mixing for curcumin delivery was described. Both the type of the non-ionic surfactant and the mixing parameters in the microfluidic system remarkably affected the characteristics of the prepared niosomes. High curcumin encapsulation of around 60% can be achieved by preparing niosomes using T85 and chol at 1:1 molar ratio. Microfluidic mixing allows the production of small and controlled size niosomes in a single step for the encapsulation of curcumin. These results will be useful for optimising the niosomes nanoparticles components to be used as a drug delivery system for curcumin and for other therapeutic agents.
